# Machine Learning in Predicting Child Malnutrition: A Meta-Analysis of Demographic and Health Surveys Data

**DOI:** 10.3390/ijerph22030449

**Published:** 2025-03-18

**Authors:** Bhagyajyothi Rao, Muhammad Rashid, Md Gulzarull Hasan, Girish Thunga

**Affiliations:** 1 Department of Applied Statistics & Data Science, Prasanna School of Public Health, Manipal Academy of Higher Education, Manipal 576104, India; bhagyajyothi.rao@manipal.edu; 2 Department of Pharmacy Practice, Manipal College of Pharmaceutical Sciences, Manipal Academy of Higher Education, Manipal 576104, India; muhammed.rashid@learner.manipal.edu (M.R.); girish.thunga@manipal.edu (G.T.); 3 Department of Pharmacotherapy, College of Pharmacy, University of Utah, Salt Lake City, UT 84112, USA; 4 Centre for Toxicovigilance and Drug Safety, Manipal College of Pharmaceutical Sciences, Manipal Academy of Higher Education, Manipal 576104, India

**Keywords:** malnutrition, childhood malnutrition, machine learning, stunting, demographic and health surveys (DHS), meta-analysis, public health

## Abstract

Background: Childhood malnutrition remains a significant global public health concern. The Demographic and Health Surveys (DHS) program provides specific data on child health across numerous countries. This meta-analysis aims to comprehensively assess machine learning (ML) applications in DHS data to predict malnutrition in children. Methods: A comprehensive search of the peer-reviewed literature in PubMed, Embase, and Scopus databases was conducted in January 2024. Studies employing ML algorithms on DHS data to predict malnutrition in children under 5 years were included. Using PROBAST (Prediction model Risk Of Bias Assessment Tool), the quality of the listed studies was evaluated. To conduct meta-analyses, Review Manager 5.4 was used. Results: A total of 11 out of 789 studies were included in this review. The studies were published between 2019 and 2023, with the major contribution from Bangladesh (*n* = 6, 55%). Of these, ten studies reported stunting, three reported wasting, and four reported underweight. A meta-analysis of ten studies reported a pooled accuracy of 68.92% (95% CI: 66.04, 71.80; I^2^ = 100%) among ML models for predicting stunting in children. Three studies indicated a pooled accuracy of 84.39% (95% CI: 80.90, 87.87; I^2^ = 100%) in predicting wasting. A meta-analysis of four studies indicated a pooled accuracy of 73.60% (95% CI: 70.01, 77.20; I^2^ = 100%) for ML models predicting underweight status in children. Conclusions: This meta-analysis indicated that ML models were observed to have moderate to good performance metrics in predicting malnutrition using DHS data among children under five years.

## 1. Introduction

Malnutrition remains a significant global health challenge, impacting millions of people across all age groups [1]. Childhood malnourishment is a major issue threatening a nation’s development [2,3]. The 2022 Joint Child Malnutrition Estimates (JME) by the United Nations Children’s Fund (UNICEF), World Health Organization (WHO), and the World Bank reveal that 10.2% (149 million) of children under 5 are stunted (low height for age). Undernutrition is linked to nearly 45% of deaths in this age group. While stunting rates have decreased slightly, wasting (low weight for height), which affects 3.1% of children, remains a significant issue, particularly in South Asia. Additionally, 2.5% of children under 5 are overweight or obese, demonstrating the multifaceted nature of malnutrition [4,5]. The global burden of malnutrition significantly and persistently affects a nation’s development, economy, society, individual health, families, and communities [6].

The DHS program offers a valuable resource for studying malnutrition. The program is funded by the U.S. Agency for International Development (USAID) and plays a critical role in improving health outcomes in developing countries. It conducts nationally representative household surveys in over 90 countries, gathering vital data on population dynamics, health, and nutrition. The information is used by governments, policymakers, NGOs, and researchers to develop and monitor health programs, track progress toward goals, and ultimately improve the lives of millions of people [3,7].

ML techniques are highly effective in handling vast quantities of data and uncovering complex patterns and relationships that traditional methods might overlook [8]. Supervised learning models, such as random forest (RF); gradient boosting machines (GBMs), including XGBoost and LightGBM; support vector machines (SVMs); k-nearest neighbors (k-NN); and logistic regression, are widely used for predicting malnutrition risk and identifying key contributing factors [9,10]. Deep learning approaches [11], including artificial neural networks (ANNs), convolutional neural networks (CNNs) for image-based assessment [8], and long short-term memory (LSTM) models for time-series analysis [12], have demonstrated high accuracy in detecting malnutrition trends. Unsupervised learning models, like K-means clustering and principal component analysis (PCA) [13], are employed to uncover hidden patterns in malnutrition datasets, while hybrid and ensemble learning techniques such as stacked ensembles and majority voting-based hybrid models combine multiple algorithms for improved predictive performance [14]. Furthermore, explainable AI (XAI) methods, including Shapley additive explanations (SHAP), local interpretable model-agnostic explanations (LIME), and feature importance ranking [15,16], provide transparency and interpretability in malnutrition prediction, making these models more actionable for healthcare practitioners and policymakers.

Integration of ML techniques with DHS data offers a powerful approach to studying and combating malnutrition, providing insights that can ultimately improve child health and well-being in developing countries [17].

However, a comprehensive synthesis of the literature on ML applications for malnutrition in young children using DHS data is notably absent. Therefore, this study aims to perform a systematic review, augmented by a meta-analysis that will help to synthesize the findings, including the types of ML techniques employed, the form of malnutrition, and performance metrics reported in the selected studies. Additionally, this review seeks to quantitatively pool the performance metrics to provide a robust estimation of the overall effectiveness of ML approaches in studying child malnutrition using DHS data. By addressing these objectives, this review aims to offer evidence-based insights into the utility, limitations, and future directions of ML to provide actionable insights that can inform healthcare practices, policy decisions, and future research directions aimed at improving the nutritional status and well-being of under-five children worldwide [1,2,18].

## 2. Materials and Methods

This systematic review was conducted following the PRISMA 2020 guidelines to ensure methodological transparency and rigor in reporting. PRISMA 2020 provides a standardized framework for the preparation and reporting of systematic review protocols, ensuring comprehensive documentation of study objectives, search strategies, selection criteria, data extraction, and synthesis methods [19].

### 2.1. Literature Search

A comprehensive literature search was conducted from inception to January 2024 to collate all the studies across three databases, including PubMed, Scopus, and EMBASE. Search terms were formulated by combining the keywords, “machine learning” “deep learning”, “Supervised Learning”, “Unsupervised Learning”, “Reinforcement Learning”, “neural network”, “Artificial Intelligence”, “Transfer Learning”, “child malnutrition”, “pediatric”, “Newborn Infants”, “Nutritional Deficiency”, “Undernutrition”, “Malnourishment”, “wasting, ” “stunting”, “underweight”, “overweight”, “micronutrients deficiency”, “Demographic and Health Surveys”, “DHS”, and their synonyms. Boolean operators “AND” and “OR” were used to develop the search strategy. The detailed search strategy for different databases is provided in Supplementary File S1.

### 2.2. Study Inclusion Criteria

English-language studies employing ML algorithms on DHS data to predict malnutrition and identify risk factors in children were included in this review. Studies that did not employ ML techniques, that used data other than DHS data, are not focused on child malnutrition, and that are reviews were excluded.

### 2.3. Study Selection Process

All identified studies from databases were retrieved to a Microsoft Excel sheet and Initial screening was performed based on titles and abstracts. The full texts of the selected studies were downloaded and screened to consider for final inclusion as per the pre-defined criteria.

### 2.4. Data Extraction

Relevant data were extracted from selected studies using a standardized data extraction sheet in Excel. Characteristics included authors of the study, year of publication, country, purpose of the study, source of data, sample size, type of malnutrition, prevalence of malnutrition addressed, missing value techniques used, train and test dataset percentage, feature selection techniques, ML algorithms used, validation methods, feature importance, key findings, best predictors of malnutrition, performance metrics, and conclusions.

### 2.5. Quality Assessment

Our study used the PROBAST (Prediction model Risk Of Bias Assessment Tool) to evaluate the risk of bias and the applicability of studies included. PROBAST features 20 signaling questions across four domains (Participants, Predictors, Outcome, and Analysis), ensuring methodological rigor and reliability in our review. All signaling questions are framed so that a “yes” response indicates no bias. If any signaling question is rated as “no” or “probably no”, it suggests a potential bias, and we must use our judgment to assess whether the domain should be rated as having a “high”, “low”, or “unclear” risk of bias [20].

The study selection, data extraction, and quality assessment were performed independently by two reviewers (BR and MGH), and any discrepancies were resolved through discussion with a third reviewer (MR/GT).

### 2.6. Data Synthesis and Statistical Analysis

All evidence collected through the systematic process was compiled into a narrative summary and is presented in the table. The data from individual studies were collected as percentages with a 95% confidence interval (CI). The percentage with the standard error was used to analyze the data and reported in terms of pooled percentage with 95% [21]. Meta-analyses were conducted separately to estimate the identified ML models’ pooled accuracy, precision, sensitivity, and other performance metrics. The I^2^ statistics were used to determine the heterogeneity; an I^2^ lower than 25% was regarded as having low heterogeneity, between 25% and 75% as having medium heterogeneity, and more than 75% as having high heterogeneity [22]. Meta-analysis was employed using Review Manager (RevMan, Version 5.4) [23]. We applied a random-effect model for data analysis and further examined the sources of heterogeneity through sensitivity analysis and subgroup analysis.

### 2.7. Sensitivity Analysis and Subgroup Analysis

The study from Papua New Guinea, which was geographically distinct from the other ten studies, was excluded from the meta-analysis during the sensitivity analysis to assess the robustness of the overall findings [24]. Subgroup analysis, focusing on specific malnutrition indicators (stunting, wasting, underweight), and ML models across the performance metrics were performed to assess the possible cause of heterogeneity [25].

### 2.8. Publication Bias

Publication bias was assessed by visually examining the distribution of effect sizes against their standard errors using a funnel plot. If the plot has a symmetrical distribution, it suggests minimal bias, while asymmetry may indicate potential publication bias. This approach helps determine the reliability of the meta-analysis findings.

## 3. Results

### 3.1. Search Results and Study Selection Process

**Figure 1:**
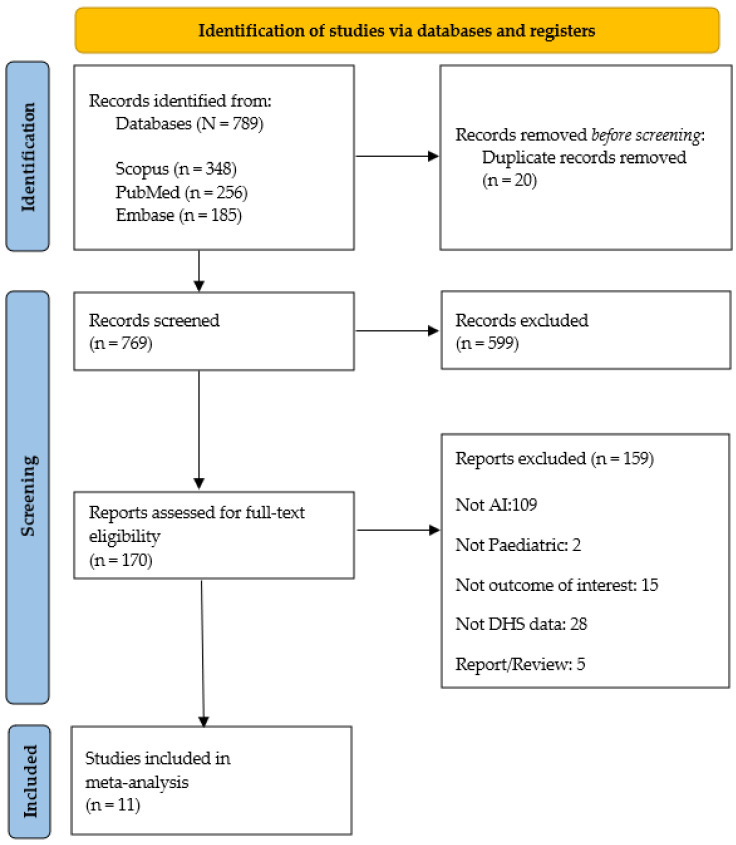
PRISMA 2020 flow diagram [36].

A total of 789 studies were retrieved from the database. Of these, 170 studies were deemed eligible for full-text screening, and ultimately 11 studies were included in the final review [7,26,27,28,29,30,31,32,33,34,35]. Studies focusing on child mortality or malnutrition in women or pregnant women, not utilizing DHS data, not employing ML algorithms, that were reviews, and published in non-English languages were the major exclusions. The detailed study selection process is depicted in the PRISMA flow diagram (Figure 1).

### 3.2. Summary of Included Study Characteristics

The included studies in our review spanning from 2019 to 2023, specifically showcasing recent advancements and applications of ML in public health, emphasizing its growing impact in the field. The studies primarily concentrate on low- and middle-income countries, especially Southeast Asia and sub-Saharan Africa. A substantial portion of this review includes Bangladesh (*n* = 6, 55%) and the African region (*n* = 4, 36%), which includes Rwanda, sub-Saharan Africa, Ethiopia, and Zambia, and additionally, as well as one study from Papua New Guinea, a geographically distinct region compared to the others in this study. Of the selected studies, 10 studies focused on stunting, with sample sizes ranging from 3380 to 9471 (mean = 6387, median = 6831, and interquartile range (IQR) = 983); 3 studies reported wasting, with sample sizes ranging from 6995 to 9471 (mean = 7848, median = 7079, and interquartile range (IQR) = 1238); 4 studies reported underweight, with sample sizes ranging from 6863 to 9471 (mean = 7602, median = 7037, and interquartile range (IQR) = 715); and there was 1 study that reported malnutrition as a whole, with sample size 56,243. All the studies utilize a cross-sectional design. The characteristics of the studies included can be found in Table 1. A detailed description of the ML algorithms used in this review is provided in Table 2. Findings from the ML models are listed in Table 3.

### 3.3. ML in Stunting Assessment

The meta-analysis pooled accuracy measures of ML models for stunting evaluation from 10 research studies. The overall pooled analysis revealed an accuracy of 68.92% (95% CI: 66.04, 71.80; I^2^ = 100%, *p* < 0.00001). Subgroup analysis demonstrated that gradient boosting achieved the highest accuracy of 73.90% (95% CI: 63.26, 84.54), followed by the Support Vector Machine (SVM) model that utilizes the Radial Basis Function (RBF) kernel while employing LASSO (Least Absolute Shrinkage and Selection Operator) regularization (LASSO-RBF support vector machine) with an accuracy of 73% (95% CI: 71.51, 74.49). Random forest exhibited a pooled accuracy of 71.77% (95% CI: 60.71, 82.84), while the support vector machine achieved an accuracy of 70.63% (95% CI: 61.62, 79.63). Other algorithms included in the analysis displayed lower accuracy. The result of the meta-analysis to pool the accuracy measures of ML models for stunting assessment is depicted in Supplementary File S2.

Four studies were incorporated into the meta-analysis to assess the pooled precision of ML models for stunting assessment. The pooled analysis yielded a precision of 67.63% (95% CI: 65.45, 69.81; I^2^ = 98%, *p* < 0.00001). Subgroup analysis revealed that gradient boosting achieved the highest precision of 72.51% (95% CI: 71.10, 73.92). This was followed by a group consisting of LASSO-RBF support vector machine, random forest with recursive feature elimination (RFE) and RBF support vector machine, random forest with RFE and conditional decision tree, all exhibiting a precision of 72% (95% CI: 70.49, 73.51). LASSO logistic regression displayed a precision of 71% (95% CI: 69.47, 72.53), while both neural networks and LASSO–conditional decision tree achieved a precision of 70% (95% CI: 68.94, 71.06). Other algorithms included in the analysis demonstrated lower precision. The result of the meta-analysis to pool the precision measures of ML models for stunting assessment is depicted in Supplementary File S3.

To evaluate the performance of ML models in identifying stunting, four studies were included in a meta-analysis to assess the pooled F1 score. The pooled analysis yielded an F1 score of 64.60% (95% CI: 60.37, 68.83; I^2^ = 100%, *p* < 0.00001). Subgroup analysis revealed that gradient boosting achieved the highest F1 score of 82.05% (95% CI: 80.83, 83.27). This was followed by naïve Bayes with an F1 score of 77.36% (95% CI: 76.03, 78.69). LASSO-RBF support vector machine displayed an F1 score of 67% (95% CI: 65.41, 68.59), while random forest with recursive feature elimination (RFE) and RBF support vector machine achieved an F1 score of 66% (95% CI: 64.41, 67.59). Other algorithms included in the analysis demonstrated lower F1 scores. The result of the meta-analysis to pool the F1 scores of ML models for stunting assessment is depicted in Supplementary File S4.

Four studies were included in a meta-analysis to assess the pooled AUC. The pooled analysis yielded an AUC of 68.68% (95% CI: 62.43, 74.92; I^2^ = 100%, *p* < 0.00001). Subgroup analysis revealed that gradient boosting achieved the highest AUC of 89% (95% CI: 88.00, 90.00). This was followed by the LASSO-RBF support vector machine with an AUC of 77% (95% CI: 75.59, 78.41). RF-RFE-RBF support vector machine had an AUC of 75% (95% CI: 73.55, 76.45), while the LASSO–conditional decision tree achieved an AUC of 74% (95% CI: 72.53, 75.47). LASSO logistic regression, RF-RFE logistic regression, and RF-RFE-XGBoost achieved an AUC of 73% (95% CI: 71.51, 74.49). LASSO-XGBoost machine had an AUC of 72% (95% CI: 70.49, 73.51). Other algorithms included in the analysis demonstrated lower AUC. The result of the meta-analysis to pool the AUC of ML models for stunting assessment is depicted in Supplementary File S5.

Meta-analysis indicated that the pooled sensitivity (recall) across the included studies is 66.43% (95% CI: 60.21, 72.66) to assess stunting. Gradient boosting has the highest sensitivity at 94.49% (95% CI: 93.76, 95.22), showing superior performance compared to other algorithms. Naïve Bayes algorithms achieved sensitivities of 83.46% (95% CI: 82.28, 84.64). Random forest and XGBoost also showed high sensitivity at 74.33% (95% CI: 53.33, 95.34) and 70.78% (95% CI: 52.88, 88.68), respectively. Logistic regression had a sensitivity of 70.22% (95% CI: 50.27, 90.18). The result of the meta-analysis to pool the sensitivities of ML models for stunting assessment is depicted in Supplementary File S6.

### 3.4. ML in Wasting Assessment

Three studies (*n* = 3) were incorporated into the meta-analysis to estimate the pooled accuracy of ML models for wasting assessment. The pooled analysis revealed an overall accuracy of 84.39% (95% CI: 80.90, 87.87; I^2^ = 100%, *p* < 0.00001). Subgroup analysis demonstrated that XGBoost achieved the highest accuracy of 88% (95% CI: 87.35, 88.65). This was followed by k-nearest neighbors with an accuracy of 87.79% (95% CI: 87.12, 88.46). The neural network model exhibited a pooled accuracy of 86.48% (95% CI: 85.59, 87.36). Finally, both support vector machine and logistic regression models displayed pooled accuracies of approximately 85%. The result of the meta-analysis to pool the accuracy of ML models for wasting assessment is depicted in Supplementary File S7.

### 3.5. ML in Underweight Assessment

Four studies (*n* = 4) were included in the meta-analysis to evaluate the pooled accuracy of ML models for assessing underweight. The pooled analysis showed an overall accuracy of 73.60% (95% CI: 70.01, 77.20; I^2^ = 100%, *p* < 0.00001). Subgroup analysis revealed that the random forest model achieved the highest accuracy at 76.24% (95% CI: 66.31, 86.18), followed by logistic regression and extreme gradient boosting, with an accuracy of 75.7%. The result of the meta-analysis to pool the accuracy of ML models for underweight assessment is depicted in Supplementary File S8.

Two studies (*n* = 2) were included in the meta-analysis to assess ML models’ pooled recall (sensitivity) for underweight evaluation. The analysis revealed an overall pooled sensitivity of 84.69% (95% CI: 79.66, 89.72; I^2^ = 100%, *p* < 0.00001). Subgroup analysis demonstrated that the linear discriminant analysis model achieved the highest sensitivity of 94.14% (95% CI: 93.59, 94.69). Logistic regression followed this with a sensitivity of 89.24% (95% CI: 80.15, 98.34), support vector machine with a sensitivity of 88.55% (95% CI: 87.81, 89.29), and random forest with a sensitivity of 85.78% (95% CI: 68.38, 103.19). The result of the meta-analysis to pool ML models’ recall (sensitivity) for underweight assessment is depicted in Supplementary File S9.

### 3.6. Identified Predictive Features

The studies reviewed highlight numerous factors contributing to child malnutrition. In 55% of the studies, the mother’s BMI and education level were identified as the most crucial predictors. Additionally, birth weight and income index were significant predictors in 35% of the studies. Other key factors influencing malnutrition in children under five include maternal age, child age, distance to water sources, maternal history of anemia, breastfeeding duration, household size, socioeconomic status, and region of residence, all of which significantly impact children’s growth and development.

### 3.7. Heterogeneity Assessment

The pooled estimates showed substantial heterogeneity (I^2^ > 75%), likely influenced by variations in dataset characteristics, including sample sizes, geographic regions, and predictor variables, which may impact the generalizability of the findings. Subgroup analysis, stratified by malnutrition indicators (stunting, wasting, underweight) and ML models across the performance metrics, revealed no significant reduction in heterogeneity, suggesting that differences in ML models were not a contributing factor.

Furthermore, sensitivity analysis was conducted by excluding one study (*n* = 10) from Papua New Guinea, a geographically distinct region compared to the other included studies. The results remained unchanged, indicating that geographic variation did not contribute to the observed heterogeneity. The forest plot of the sensitivity analysis with 10 studies is in the Supplementary File S10. The asymmetry in the funnel plot suggests a possible publication bias. The funnel plot for stunting accuracy across ML models is in the Supplementary File S11.

### 3.8. Risk of Bias Assessment

The risk of bias assessment revealed that most studies exhibited a low risk of bias across the four evaluated domains, indicating strong methodological rigor in study design and implementation. In the “participants” domain, the majority of studies utilized appropriate data sources with well-defined inclusion and exclusion criteria, minimizing selection bias. Similarly, the “predictors” domain demonstrated consistency in defining and assessing predictive variables, ensuring their availability at the time of model application. The “outcome” domain, while generally well-defined, exhibited some variability, as a subset of studies lacked expected results, introducing potential bias. The “analysis” domain showed a certain amount of variability, with few studies encountering challenges related to outcome selection, sample size limitations, and methodological inconsistencies, which may impact the reliability of the findings.

Despite these variations, the overall risk of bias was low in most studies, reinforcing the robustness and reliability of the included research. The final risk of bias rating was determined based on assessments across all four domains. Studies demonstrated a low risk of bias in the participants and predictor domains, reflecting methodological rigor in study design and implementation. However, inconsistencies in outcome determination and statistical analysis contributed to heterogeneity, potentially affecting the generalizability of findings. A detailed breakdown of the risk of bias assessment is provided in the Supplementary File S12.

## 4. Discussion

This review reveals significant insights into applying various ML techniques in addressing child malnutrition in different forms. Supervised learning strategies played a significant role in the selected studies due to their ease of interpretation and simplicity. Logistic regression was often employed for binary classification tasks, such as determining whether nutritional signs like stunting, wasting, and underweight were present or absent [37,38,39]. We observed that both decision trees and random forests were widely used. Decision trees offer an easy-to-understand visual depiction of decision rules, while random forests, an ensemble technique, combine several decision trees to increase prediction accuracy and decrease overfitting [17,34,40]. Hyperplanes are used by support vector machines (SVMs) to divide classes in classification [41,42,43]. Studies by Shahriar et al., 2019 [33] and Abid et al., 2021 [31] using artificial neural networks (ANNs) to study malnutrition in children provide high prediction accuracy by modelling complex, nonlinear relationships in data, which is essential for identifying at-risk children. A study by Khan and Yunus [28] aimed to develop a majority voting-based hybrid ensemble (MVBHE) learning model to enhance prediction accuracy by incorporating bagging, boosting, and voting algorithms. Meta-analysis revealed that ensemble ML models and tree-based models are more effective for this prediction task than linear models and other techniques [44,45,46]. The models were developed using distinct training and validation datasets and validated using 5-, 10-, or k-fold cross-validation. Some studies utilized RFE, LASSO [27], and the chi-square test [7] for dimensionality reduction, ensuring that only the most relevant features were retained for model training and evaluation. The performance of ML models developed for predicting child malnutrition included in the current study was observed to have an acceptable range of performance metrics such as accuracy, precision, recall, and F1 score, with AUC as a key indicator of their effectiveness.

We examined various feature selection techniques used in the reviewed studies, which included Gini importance, permutation importance, and chi-square to select the relevant features for this study. Studies mentioned the usage of SHAP, mean decrease Gini, and the feature importance ranking technique to ensure the interpretability of the model. Model interpretability is essential for ensuring transparency, trust, and real-world usability in ML-based malnutrition prediction [47]. While traditional models like logistic regression are inherently interpretable, advanced models such as random forest, XGBoost, and neural networks often act as black-box models, limiting their adoption in public health decision-making. XAI techniques like SHAP and LIME (local interpretable model-agnostic explanations) are best for identifying the most significant factors of malnutrition [48]. Integrating ML models with XAI methods revealed key predictors such as maternal nutrition, birth weight, household income, dietary diversity, and sanitation, which consistently influenced malnutrition risk assessments across different ML models.

One of this study’s major strengths is that it includes studies from nationally representative DHS datasets. These large datasets provide comprehensive information on various health, socioeconomic, and demographic variables, enabling robust model training and validation. Comparative performance evaluations of multiple ML algorithms enhance the reliability of the findings, showcasing the potential of these techniques to improve malnutrition prediction.

To the best of our knowledge, this is the first meta-analysis on the application of ML in predicting child malnutrition using DHS data. However, there are reviews on predictors of stunting among children under five years of age [2], artificial intelligence in malnutrition [49], meta-analyses of the global leadership initiative on malnutrition criteria [50], prevalence and predictors of stunting [1], bibliometric analysis on data mining and ML techniques applied to public health problems [18], etc.

While this study provides valuable insights, it has a few limitations. The variation in datasets, including differences in sample sizes, geographic regions, and predictor variables, may have contributed to high heterogeneity, potentially impacting the generalizability of the findings. Therefore, results should be interpreted with caution. Although machine learning models demonstrate high predictive accuracy, their complexity poses challenges in interpretability, making it difficult to pinpoint the key drivers of malnutrition—an essential aspect for effective policymaking.

Moreover, this study relies on secondary DHS data, which may not fully capture real-time, region-specific factors influencing malnutrition. The analysis is primarily focused on low- and middle-income countries (LMICs), particularly Bangladesh and sub-Saharan Africa, limiting its applicability to high-income countries (HICs) or other LMICs with different socioeconomic conditions and healthcare systems. Additionally, while this study employed a comprehensive search strategy across three major databases (Scopus, PubMed, and Embase), some relevant studies may have been omitted. The exclusion of non-English publications, conference proceedings, and grey literature further restricts the scope. Despite these limitations, this study contributes to the growing evidence on the application of ML in malnutrition research, offering important directions for future studies and policy interventions.

Malnutrition is influenced by a complex interplay of spatial and environmental factors. While socioeconomic gradients in regional health inequalities are well documented, the interaction between socioeconomic deprivation and climate vulnerability, especially in nearby neighborhoods, and its impact on health disparities using ML methods remains less explored [42,51,52,53,54]. This highlights the need for a deeper regional understanding to address the spatial spillover effects and the nonlinear influence of covariates on childhood malnutrition. This can be effectively tackled through specialized spatial ML techniques by incorporating spatial lag components in the model. Overweight and wasting among children are also significant global public health issues that seem to be less explored compared to stunting. Wasting in Indian children is a serious issue, with India having the highest child-wasting rate in the world [55,56]. Childhood obesity in India is a pressing public health concern [57]. Due to the growing prevalence and important consequences of wasting and obesity, it is vital to use comprehensive data like DHS and advanced analytical methods like ML to inform effective public health strategies and interventions to combat these problems to improve individual health outcomes and yield broader societal and economic benefits.

## 5. Conclusions

ML techniques provide powerful tools for predicting child malnutrition, with ensemble and tree-based models delivering the highest accuracy. Despite challenges like model interpretability and data variability, these advanced computational methods show great potential for enhancing public health strategies and outcomes. Tackling regional health inequalities also requires consideration of spatial spillover and the nonlinear impact of covariates on childhood malnutrition, which ML techniques are well suited to address. Ongoing research and collaboration are essential to fully leverage these technologies in the global fight against child malnutrition.

## Figures and Tables

**Table 1:** Characteristics of included studies.

Reference	Country	Purpose of Study	Source of Data	Form of Malnutrition Addressed	Prevalence	Missing Value Imputation
[26]	Rwanda	Predicting childhood stunting	RDHS (2019–2020)	Stunting	33.35%	K-nearest-neighbors imputer
[27]	Papua New Guinea	Predicting stunting and the key predictors	PNG DHS (2016–2018)	Stunting	39.70%	Missing indicator method (MIM)
[28]	Sub-Saharan Africa	Predicting malnutrition	DHS data from 14 SSA countries	Stunting	Malnourished 39.3%	Manual elimination of missing/irrelevant information
[29]	Ethiopia	Predicting malnutrition	EDHS-2016	Stunting Wasting Underweight	(Stunted 38.4%) (Wasted 10%) (Underweight 23.3%) (Malnutrition 46.6%)	NR
[30]	Zambia	Predicting childhood stunting	ZDHS-2018	Stunting	34.2%	Missing instances were dropped
[31]	Bangladesh	Predicting stunting and the key predictors	BDHS-2014	Stunting	36.4%	Filtering procedure
[7]	Bangladesh	Predicting malnutrition and the key predictors	BDHS-2014	Stunting Wasting Underweight	(Stunted 35.4%) (Wasted 5.4%) (Underweight 32.8%)	NR
[32]	Bangladesh	Predicting stunting and interaction between the predictors	BDHS-2014	Stunting	36.50%	Excluded the missing values
[33]	Bangladesh	Predicting malnutrition with the ANN approach	BDHS-2014	Stunting Wasting Underweight	NR	NR
[34]	Bangladesh	Predicting childhood stunting	BDHS-2014	Stunting	36.18%	Excluded the missing values
[35]	Bangladesh	Predicting childhood malnutrition	BDHS-2014	Underweight	32.90%	Excluded the missing values

RDHS—Rwanda Demographic and Health Survey; PNG DHS—Papua New Guinea Demographic Health Survey; EDHS—Ethiopian Demographic and Health Survey; ZDHS—Zambia Demographic and Health Survey; BDHS—Bangladesh Demographic Health Survey.

**Table 2:** ML and DL models and performance metrics employed.

Reference	Sample Size	Train and Test (%)	Feature Selection	ML Algorithms	Performance Metrics	Method of Validation	Feature Importance
[26]	3814	NR	Chi-square and SMOTE for balancing data	Support vector machines, naïve Bayes, random forest, logistic regression, and extreme gradient boosting	Confusion matrix, receiver operating characteristic curve, accuracy, sensitivity, specificity, F1 score, and area under the curve (AUC).	10-fold cross-validation	NR
[27]	3380	Training 90% and test 10%	Embedded LASSO and the wrapped random forest–recursive feature elimination (RF-RFE)	Logistic regression, a conditional decision tree, a support vector machine with a radial basis function kernel, and an extreme gradient boosting machine (XGBoost)	AUC, accuracy, precision, recall, F1 score	10-fold cross-validation	Shapley additive explanations (SHAP)
[28]	56,243	Training 80% and test 20%	Gini importance	The research used bagging, boosting, and voting on random forest, decision tree, extreme gradient boosting, and k-nearest neighbors to generate the MVBHE model.	Accuracy, precision, recall, and the F1 score	10-fold cross-validation	NR
[29]	9471	Training 70% and test 30%	Based on retrospective information	XGBoost, generalized linear model (GLM), NNet, RF, k-NN) using ‘Stacking’	Confusion matrix, prediction, accuracy, sensitivity, and specificity	10-fold cross-validation	Mean decrease Gini
[30]	6799	Training 70% and test 30%	Random forest	Logistic regression, random forest, support vector machine (SVM), naïve Bayes, and extreme gradient boosting (XGBoost)	Accuracy, recall, sensitivity, specificity, precision, F1 score, Cohen’s kappa, and area under the curve (AUC)	3-fold cross-validation	Random forest
[31]	7256	Training 70% and test 30%		Decision tree algorithm, Support vector machine (SVM) and artificial neural network (ANN)	Precision, recall, F1 score	N/A	
[7]	7079	NR	Chi-square analysis	Support vector machine (SVM), random forest (RF), and LR	Accuracy and area under the curve (AUC)	10-fold CV	NR
[32]	6170	NR	Based on the literature review and pre-analysis	Classification tree, ensemble of trees	Accuracy, sensitivity, specificity, and the area under the receiver operating characteristic curve (ROC)	10-fold cross-validation	NR
[33]	6995	Training 90% and test 10%	Literature review	Support vector machine (SVM) classifier, decision tree classifier, naïve Bayes classifier, and random forest classifier besides the artificial neural network (ANN)	Accuracy	10-fold cross-validation	Backward elimination on the predictive model
[34]	6044	Training 66.67% and test 33.33%	Bivariate analysis, logistic regression model with stepwise variable selection	Gradient boosting, random forests, support vector machines, classification tree, logistic regression, linear discriminant analysis, neural network, regularized discriminant analysis, and logistic regression	Sensitivity, specificity, area under the receiver operating characteristic curve (AUC), and F-measure	3-fold cross-validation	NR
[35]	6863	Training 75% and test 25%	Chi-square analysis	Linear discriminant analysis, K-nearest neighbors, support vector machines, random forest, and logistic regression	Accuracy, sensitivity, specificity, and Cohen’s k statistic	10-fold cross-validation	RF feature selection

**Table 3:** Findings from ML and DL models.

Reference	Findings	Best Predictive Features	Conclusion	Software/Tool Used
[26]	Gradient boosting classifier significantly outperformed other methods, identifying stunted children at 79.33% accuracy.	Mother’s height, water source distance, child’s age, birth weight, anemia history.	Model can help detect early stages of stunting and wasting.	Python as statistical software.
[27]	LASSO-XGB combined model provided best predictions.	Highlands region, age of the child, breastfeeding duration, maternal BMI.	Combining LASSO and XGBoost best predicted outcomes.	Data processing: STATA 17.0, Analyses: RStudio 4.1.2
[28]	Random forest algorithm had the highest accuracy.	Mother’s age, income index, birth order, child’s weight, anemia history.	The MVBHE model is recommended for its accuracy and robustness.	SPSS version 26 for experimental run.
[29]	XGBTree algorithm worked best for stunting and wasting.	Time to water source, anemia history, child birth weight, mother’s education level.	Findings support improvement in access to clean water and maternal education.	The R programming language (version 3.6.0).
[30]	Random forest was the best performing model for the dataset.	Child’s and mother’s social and economic features.	Study demonstrates potential of machine learning in health outcome prediction.	Python version 3.10.2.
[31]	Decision tree accuracy was 74%, SVM was 72%, and KNN was 69%.	Mother’s highest education level, child’s age, birth order, child’s weight.	Addressing demographic, socioeconomic, and nutritional factors can improve outcomes.	SPSS version 23.0 for data cleaning.
[7]	RF accurately classified stunting, wasting, and underweight categories.	Region, child’s age, father’s education, mother’s BMI.	Identification and prediction of childhood malnutrition using RF.	STATA version 14 and R i386 4.0.0.
[32]	Decision tree rules yielded more accurate results compared to other models.	Wealth, area and division of residence, mother’s education level.	Tailored interventions based on socioeconomic and demographic factors are needed.	R (version 3.6.0).
[33]	ANN approach showed best results with accuracy higher than other models.	Residence, sex of the child, father’s education, mother’s BMI, household size.	Deep learning can effectively determine malnutrition status.	Python “numpy” library, Tensorflow (https://www.tensorflow.org), “Keras”.
[34]	GBOOST had the highest accuracy among the methods evaluated.	Child age, wealth index, maternal education, previous birth interval.	ML can support building accurate prediction models for malnutrition.	NR
[35]	RF algorithm demonstrated the best performance for classification tasks.	Child’s age, mother’s education, wealth index, mother’s BMI.	Recommends RF classification with RF regression for precise results.	NR

## Data Availability

The data used in this article are not deposited in any repository. However, they can be made available upon request (to the corresponding author).

## Supplementary Materials

The following supporting information can be downloaded at: https://www.mdpi.com/article/10.3390/ijerph22030449/s1, Supplementary Files S1–S12: The detailed search strategy in different databases.
