# Supplementary material for: Machine Learning in Predicting Child Malnutrition: A Meta-Analysis of Demographic and Health Surveys Data
Source: Int J Environ Res Public Health. 2025 Mar 18;22(3):449. doi: 10.3390/ijerph22030449 (PMC11941938; doi:10.3390/ijerph22030449)
Supplement: Supplementary file 1 [file ijerph-22-00449-s001.zip › File S1.pdf]

Supplemental File S1. The detailed search strategy in different databases.

A. PubMed Search Strategy

Search	Query	Items found
#1	((((((((((children) OR (child)) OR (pediatric)) OR (Infants, Newborn)) OR (Newborn Infant)) OR (Newborn Infants)) OR (Newborns)) OR (Newborn)) OR (Neonate)) OR (Neonates)) OR (baby)	42,07,906
#2	((((((((((((((Nutritional Deficiency) OR (Nutritional Deficiencies)) OR (Undernutrition)) OR (Malnourishment)) OR (Malnourishment)) OR (malnutrition)) OR (anemia)) OR (wasting)) OR (stunting)) OR (underweight)) OR (overweight)) OR (micronutrients deficiency)) OR (micronutrient deficiency)) OR (obesity)) OR (malnourished)	10,76,411
#3	((((((((((Machine learning) OR (DeepLearning)) OR (Deep Learning)) OR (Supervised Learning)) OR (Unsupervised Learning)) OR (Reinforcement Learning)) OR (neural network)) OR (Intelligence, Artificial)) OR (Transfer Learning)) OR (Learning, Machine)) OR (Learning, Transfer)	5,29,584
#4	(((National Family Health Survey) OR (NFHS)) OR (demographic and Health Survey)) OR (demographic health survey)	19,48,052
#5	(((#1) AND (#2)) AND (#3)) AND (#4)	256

B. SCOPUS Search Strategy

Search	Query	Items found
#1	(ALL ("children") OR ALL ("child") OR ALL ("pediatric") OR ALL ("newborn infant") OR ALL ("newborn infants") OR ALL ("newborns") OR ALL ("newborn") OR ALL ("neonate") OR ALL ("neonates") OR ALL("baby"))	9,857,515
#2	(ALL ("nutritional deficiency") OR ALL ("nutritional deficiencies") OR ALL ("undernutrition") OR ALL ("malnourishment") OR ALL ("malnutrition") OR ALL ("anemia") OR ALL ("stunting") OR ALL ("underweight") OR ALL ("overweight") OR ALL ("micronutrients deficiency") OR ALL ("micronutrient deficiency") OR ALL ("obesity") OR ALL ("malnourished") OR ALL ("wasting"))	2,722,127
#3	(ALL ("machine learning") OR ALL ("deep learning") OR ALL ("deep learning") OR ALL ("supervised learning") OR ALL ("unsupervised learning") OR ALL ("reinforcement learning") OR ALL ("neural network") OR ALL ("artificial intelligence") OR ALL ("transfer learning"))	6,025,568
#4	(ALL ("national and family health survey") OR ALL ("nfhs") OR ALL ("demographic and health survey") ORALL ("demographic health survey"))	62,367
#5	((ALL ("national and family health survey") OR ALL ("nfhs") OR ALL ("demographic and health survey") ORALL ("demographic health survey"))) AND ((ALL ("machine learning") OR ALL ("deeplearning") OR ALL ("deep learning") OR ALL ("supervised learning") OR ALL ("unsupervised learning") OR ALL ("reinforcementlearning") OR ALL ("neural network") OR ALL ("artificial intelligence") OR ALL ("transfer learning"))) AND ((ALL ("nutritional deficiency") OR ALL ("nutritional deficiencies") OR ALL ("undernutrition") OR ALL ("malnourishment") OR ALL ("malnutrition") OR ALL ("anemia") OR ALL ("stunting") OR ALL ("underweight")OR ALL ("overweight") OR ALL ("micronutrients deficiency") OR ALL ("micronutrient deficiency") OR ALL ("obesity") OR ALL ("malnourished") OR ALL ("wasting"))) AND ((ALL ("children") OR ALL ("child") OR ALL ("pediatric") OR ALL ("newborn infant") OR ALL ("newborn infants") OR ALL ("newborns") OR ALL ("newborn") OR ALL ("neonate") OR ALL ("neonates") OR ALL ("baby")))	348

C. Embase Search Strategy

Search	Query	Items found
#1	(‘machine learning' OR 'deep learning' OR 'supervised learning' OR 'unsupervised learning' OR 'reinforcement learning ' OR ' neural network' OR ' artificial intelligence' OR 'transfer learning') AND ('national family health survey 'OR 'nfhs' OR 'demographic health survey' OR 'demographic and health survey') AND ('malnutrition' OR 'malnourishment' OR 'nutritional deficiencies' OR ' underweight ' OR ' overweight ' OR ' stunting ' OR 'nutritional deficiencies' OR ' wasting ' OR ' overweight ') AND ('child' OR ' children ' OR ' pediatric ' OR ' newborn infant ' OR ' newborns ' OR ' neonate ' OR ' baby ' OR ' newborns ' OR ' neonate ')	185
