# Supplementary material for: Machine Learning in Predicting Child Malnutrition: A Meta-Analysis of Demographic and Health Surveys Data
Source: Int J Environ Res Public Health. 2025 Mar 18;22(3):449. doi: 10.3390/ijerph22030449 (PMC11941938; doi:10.3390/ijerph22030449)
Supplement: Supplementary file 1 [file ijerph-22-00449-s001.zip › File S12.pdf]

Assessment of Risk of Bias and Concerns Regarding Applicability using PROBAST (Prediction model Risk Of Bias Assessment Tool)

PROBAST is structured as four key domains. Each domain is judged for risk of bias (low, high, or unclear) and includes signaling questions to help make judgments. Signaling questions are rated as yes (Y), probably yes (PY), probably no (PN), no (N), or no information (NI). All signaling questions are phrased so that "yes" indicates the absence of bias. Any signaling question rated as "no" or "probably no" flags the potential for bias; we need to use our judgment to determine whether the domain should be rated as "high", "low" or "unclear" risk of bias.

1. **Participants**
   - Were appropriate data sources used, e.g., cohort, RCT, or nested case-control study data?
   - Were all inclusions and exclusions of participants appropriate?

2. **Predictors**
   - Were predictors defined and assessed in a similar way for all participants
   - Were predictor assessments made without knowledge of outcome data?
   - Are all predictors available at the time the model is intended to be used?

3. **Outcome**
   - Was the outcome determined appropriately?
   - Was a pre-specified or standard outcome definition used?
   - Were predictors excluded from the outcome definition?
   - Was the outcome defined and determined in a similar way for all participants?
   - Was the outcome determined without knowledge of predictor information?
   - Was the time interval between predictor assessment and outcome determination appropriate?

4. **Analysis**
   - Were there a reasonable number of participants with the outcome?
   - Were continuous and categorical predictors handled appropriately?
   - Were all enrolled participants included in the analysis?
   - Was selection of predictors based on univariable analysis avoided?
   - Were complexities in the data (e.g. censoring, competing risks, sampling of controls) accounted for appropriately?
   - Were relevant model performance measures evaluated appropriately?
   - Were model overfitting and optimism in model performance accounted for?
   - Do predictors and their assigned weights in the final model correspond to the results from multivariable analysis?

Overall Judgement

Low risk of bias

- If all domains were rated low risk of bias.
- If a prediction model is developed without any external validation, and it is rated as low risk of bias for all domains, consider downgrading to high risk of bias. Such a model can only be considered as low risk of bias if the development is based on a very large data set and includes some form of internal validation.

High risk of bias

If at least one domain is judged to be at high risk of bias.

Unclear risk of bias

If an unclear risk of bias was noted in at least one domain and it was low risk for all other domains.

Quality assessment of included studies

Risk of bias assessment

Study	ROB				Applicability			Overall	
	Participants	Predictors	Outcome	Analysis	Participants	Predictors	Outcome	ROB	Applicability
Bitew et al., 2022 [1]	+	+	+	+	+	+	+	+	+
Chilyabanyama et al., 2022 [2]	+	+	+	-	+	+	-	-	+
Khan et al., 2021 [3]	+	+	+	+	+	+	+	+	+
Mansur et al., 2021 [4]	+	+	-	-	+	+	-	-	-
Abid et al., 2021 [5]	+	+	+	+	+	+	+	+	+
Ndagijimana et al., 2023 [6]	+	+	+	+	+	+	+	+	+
Rahman et al., 2021 [7]	+	+	+	-	+	+	-	-	+
Shen et al., 2023 [8]	+	+	+	+	+	+	+	+	+
Shahriar et al., 2019 [9]	+	+	-	-	+	+	-	-	-
Khan et al., 2023 [10]	+	+	+	+	+	+	+	+	+
Talukder et al., 2020 [11]	+	+	-	+	+	+	-	-	-

ROB = risk of bias.
+ indicates low ROB/low concern regarding applicability.
- indicates high ROB/high concern regarding applicability.
? indicates unclear ROB/unclear concern regarding applicability.

References

1. Bitew, F.H.; Sparks, C.S.; Nyarko, S.H. Machine learning algorithms for predicting undernutrition among under-five children in Ethiopia. *Public Health Nutr.* **2022**, *25*, 269–280. https://doi.org/10.1017/S1368980021004262.
2. Chilyabanyama, O.N.; Chilengi, R.; Simuyandi, M.; Chisenga, C.C.; Chirwa, M.; Hamusonde, K.; Saroj, R.K.; Iqbal, N.T.; Ngaruye, I.; Bosomprah, S. Performance of Machine Learning Classifiers in Classifying Stunting among Under-Five Children in Zambia. *Children* **2022**, *9*, 1082. https://doi.org/10.3390/children9071082.
3. Khan, J.R.; Tomal, J.H.; Raheem, E. Model and variable selection using machine learning methods with applications to childhood stunting in Bangladesh. *Inform. Health Soc. Care* **2021**, *46*, 425–442. https://doi.org/10.1080/17538157.2021.1904938.

4. Mansur, M.; Afiaz, A.; Hossain, M.S. Sociodemographic risk factors of under-five stunting in Bangladesh: Assessing the role of interactions using a machine learning method. *PLoS ONE* **2021**, *16*, e256729. https://doi.org/10.1371/journal.pone.0256729.
5. Abid, M.H.; Haque, A.; Kamrul Hossain, M. *Factors Causing Stunting Among Under-Five Children in Bangladesh*; Springer: Singapore, 2021; Volume 1183. https://doi.org/10.1007/978-981-15-5856-6_5.
6. Ndagijimana, S.; Kabano, I.H.; Masabo, E.; Ntaganda, J.M. Prediction of Stunting among Under-5 Children in Rwanda Using Machine Learning Techniques. *J. Prev. Med. Public Health* **2023**, *56*, 41–49. https://doi.org/10.3961/jpmph.22.388.
7. Rahman, S.M.J.; Ahmed, N.A.M.F.; Abedin, M.M.; Ahammed, B.; Ali, M.; Rahman, M.J.; Maniruzzaman, M. Investigate the risk factors of stunting, wasting, and underweight among under-five Bangladeshi children and its prediction based on machine learning approach. *PLoS ONE* **2021**, *16*, e0253172. https://doi.org/10.1371/journal.pone.0253172.
8. Shen, H.; Zhao, H.; Jiang, Y. Machine Learning Algorithms for Predicting Stunting among Under-Five Children in Papua New Guinea. *Children* **2023**, *10*, 1638. https://doi.org/10.3390/children10101638.
9. Shahriar, M.M.; Iqubal, M.S.; Mitra, S.; Das, A.K. A deep learning approach to predict malnutrition status of 0–59 Month's Older Children in Bangladesh. In Proceedings of the 2019 IEEE International Conference on Industry 4.0, Artificial Intelligence, and Communications Technology (IAICT), Bali, Indonesia, 1–3 July 2019; pp. 145–149.
10. Khan, M.N.A.; Yunus, R.M. A hybrid ensemble approach to accelerate the classification accuracy for predicting malnutrition among under-five children in sub-Saharan African countries. *Nutrition* **2023**, *108*, 111947. https://doi.org/10.1016/j.nut.2022.111947.
11. Talukder, A.; Ahammed, B. Machine learning algorithms for predicting malnutrition among under-five children in Bangladesh. *Nutrition* **2020**, *78*, 110861. https://doi.org/10.1016/j.nut.2020.110861.
