# Supplementary figures and images for: Machine Learning in Predicting Child Malnutrition: A Meta-Analysis of Demographic and Health Surveys Data
Source: Int J Environ Res Public Health. 2025 Mar 18;22(3):449. doi: 10.3390/ijerph22030449 (PMC11941938; doi:10.3390/ijerph22030449)

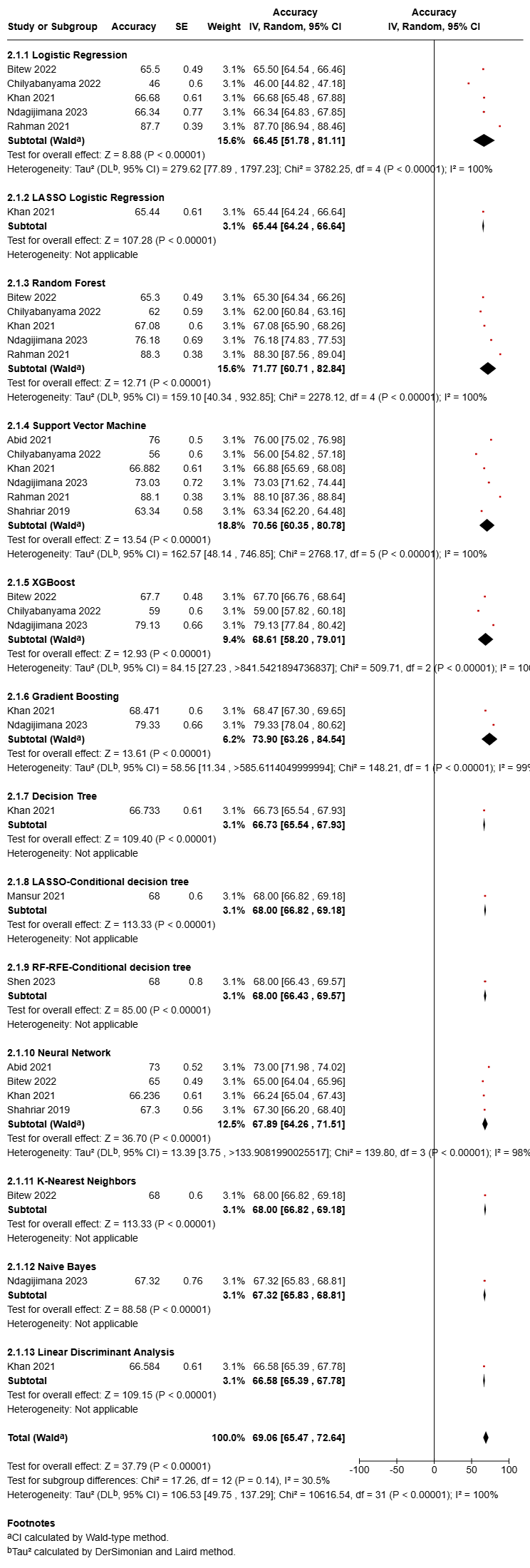

Supplement: Supplementary file 1 [file ijerph-22-00449-s001.zip › File S10.png]

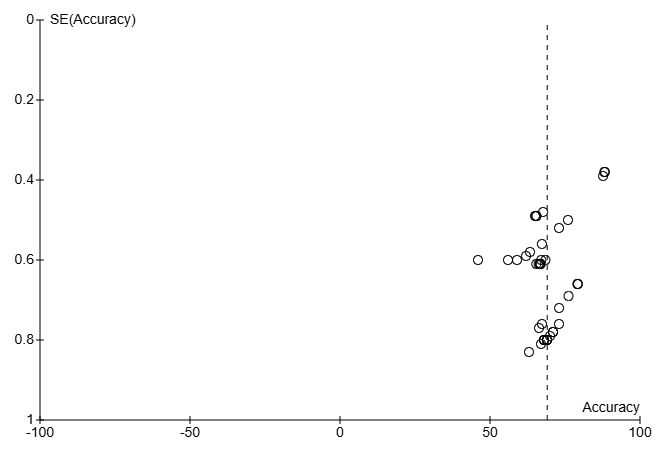

Supplement: Supplementary file 1 [file ijerph-22-00449-s001.zip › File S11.png]

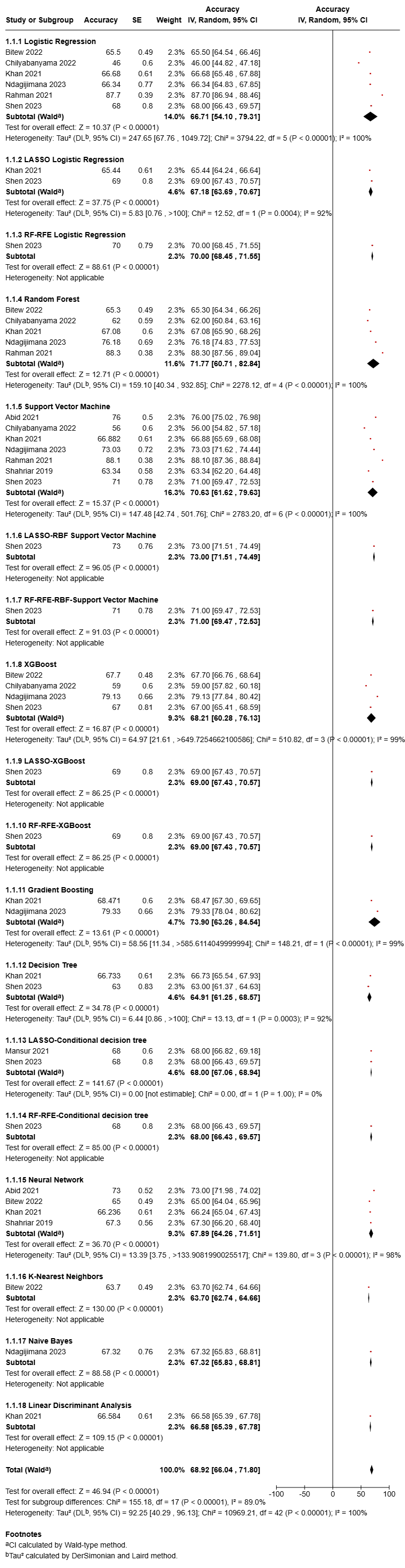

Supplement: Supplementary file 1 [file ijerph-22-00449-s001.zip › File S2.png]

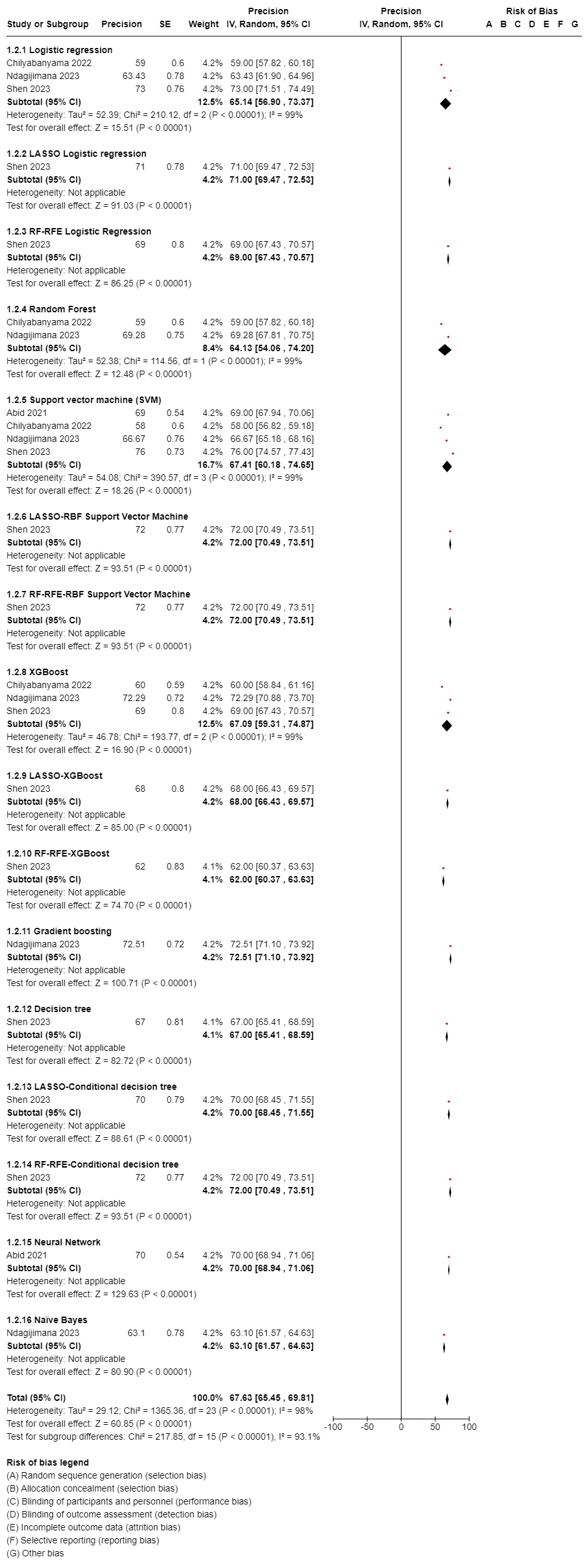

Supplement: Supplementary file 1 [file ijerph-22-00449-s001.zip › File S3.png]

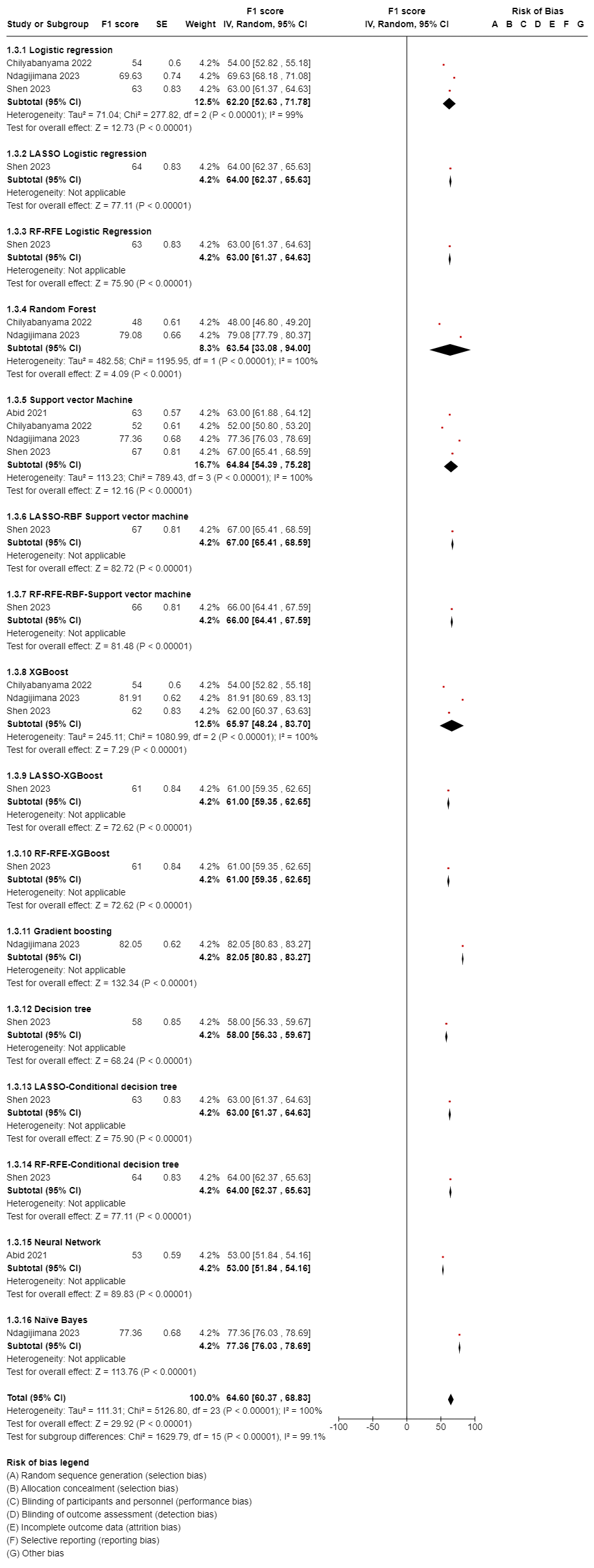

Supplement: Supplementary file 1 [file ijerph-22-00449-s001.zip › File S4.png]

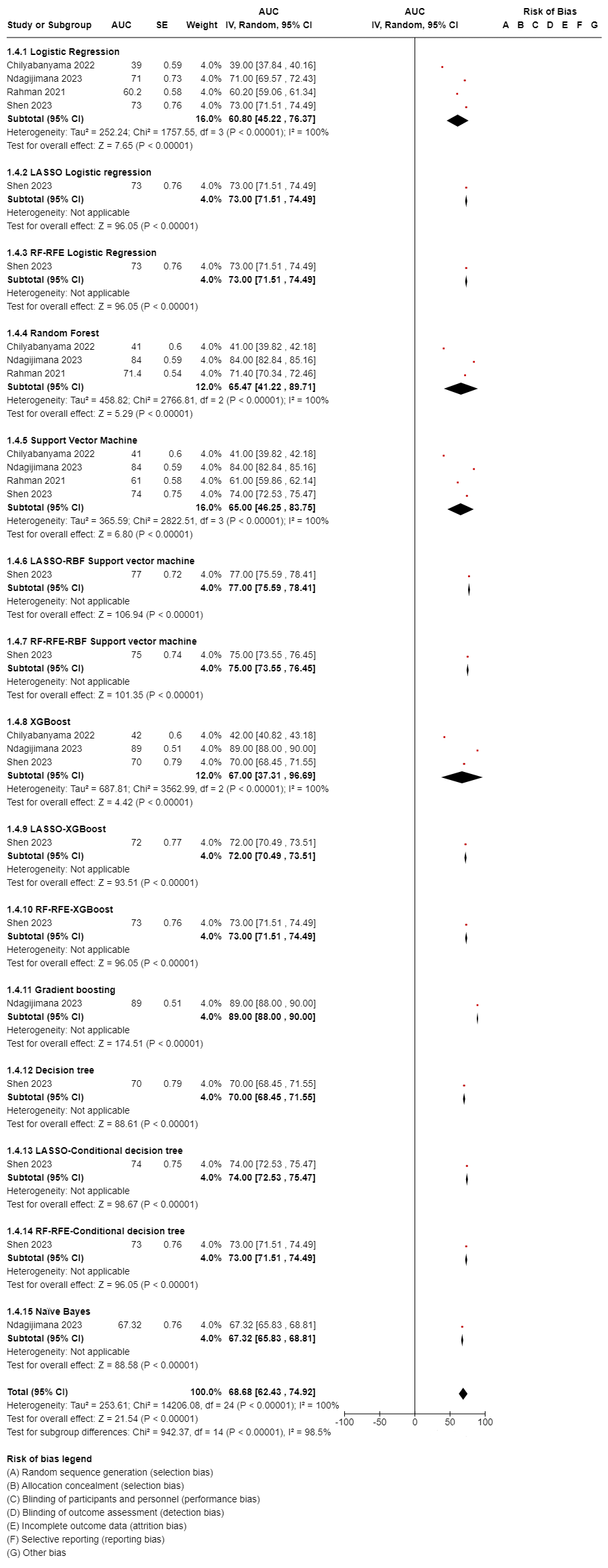

Supplement: Supplementary file 1 [file ijerph-22-00449-s001.zip › File S5.png]

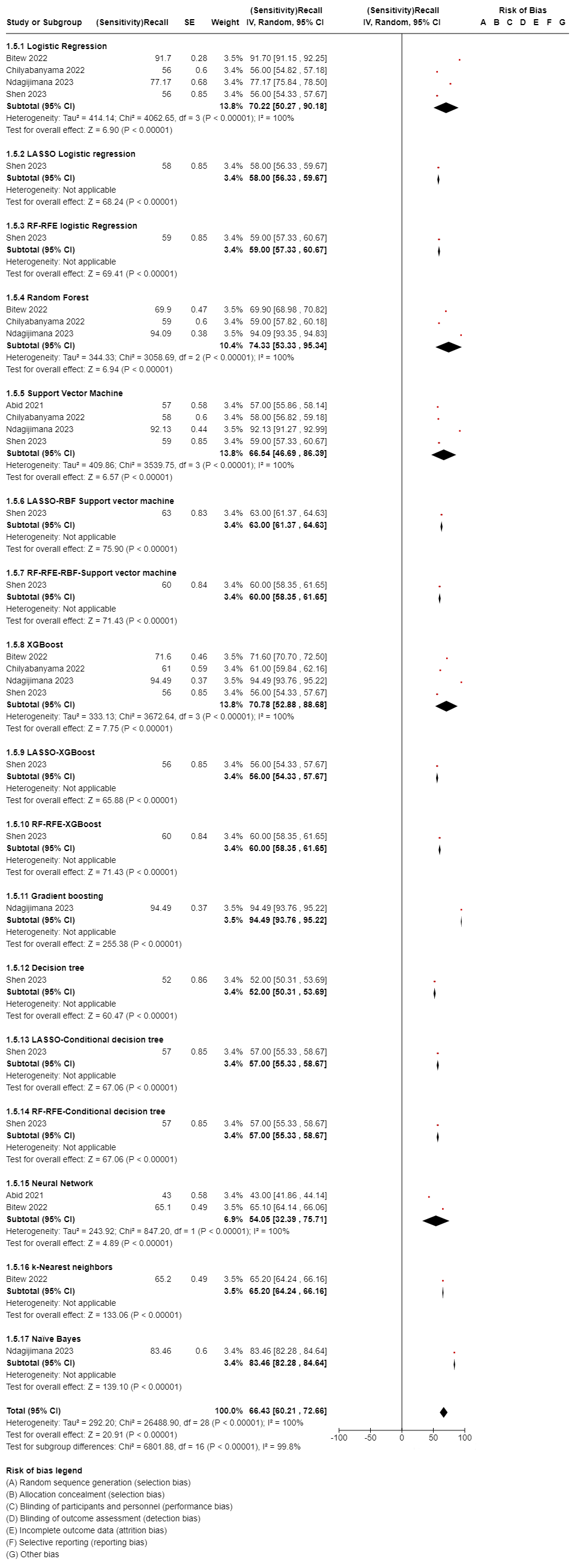

Supplement: Supplementary file 1 [file ijerph-22-00449-s001.zip › File S6.png]

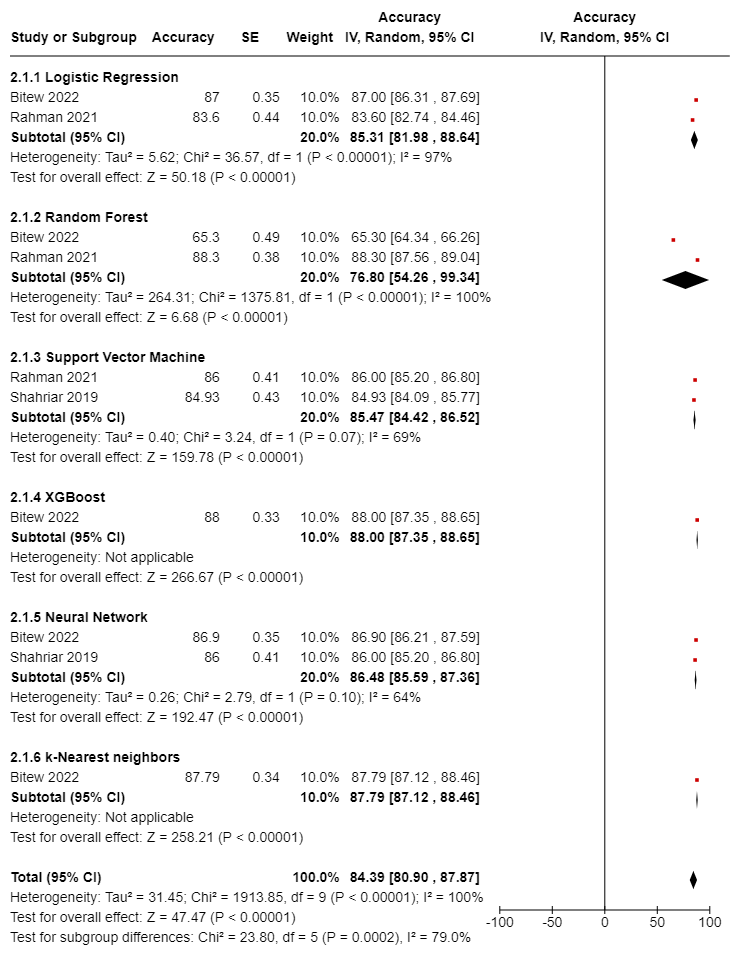

Supplement: Supplementary file 1 [file ijerph-22-00449-s001.zip › File S7.png]

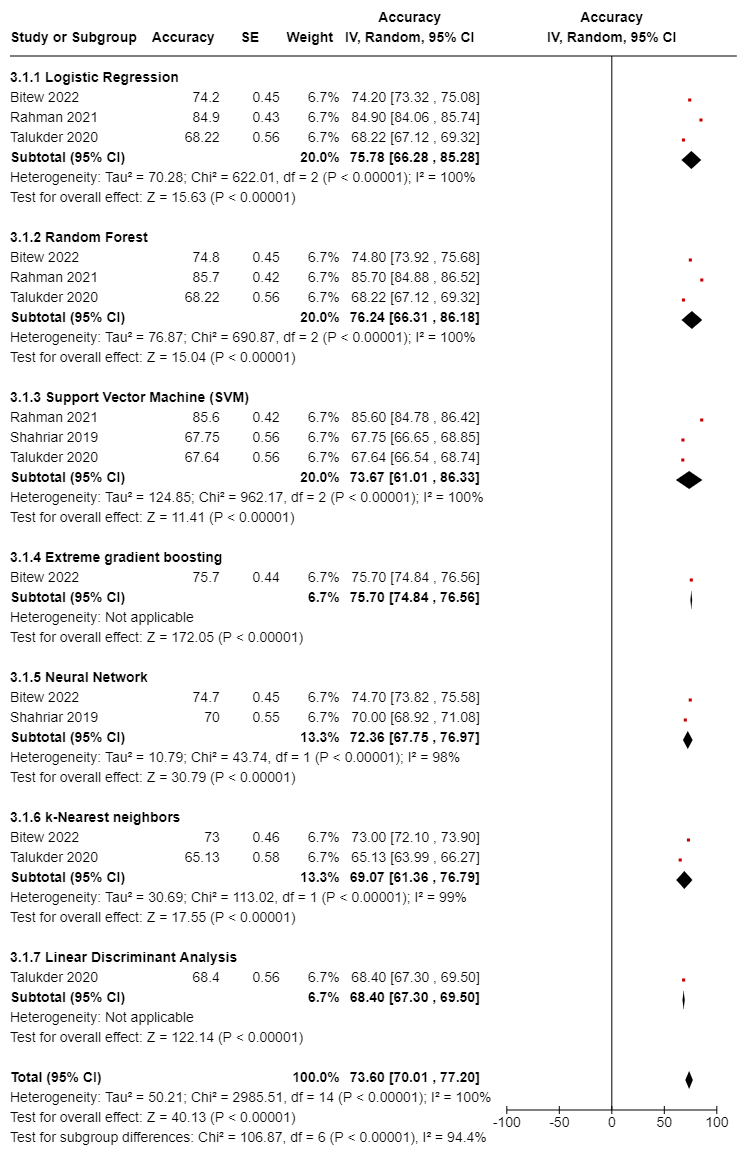

Supplement: Supplementary file 1 [file ijerph-22-00449-s001.zip › File S8.png]

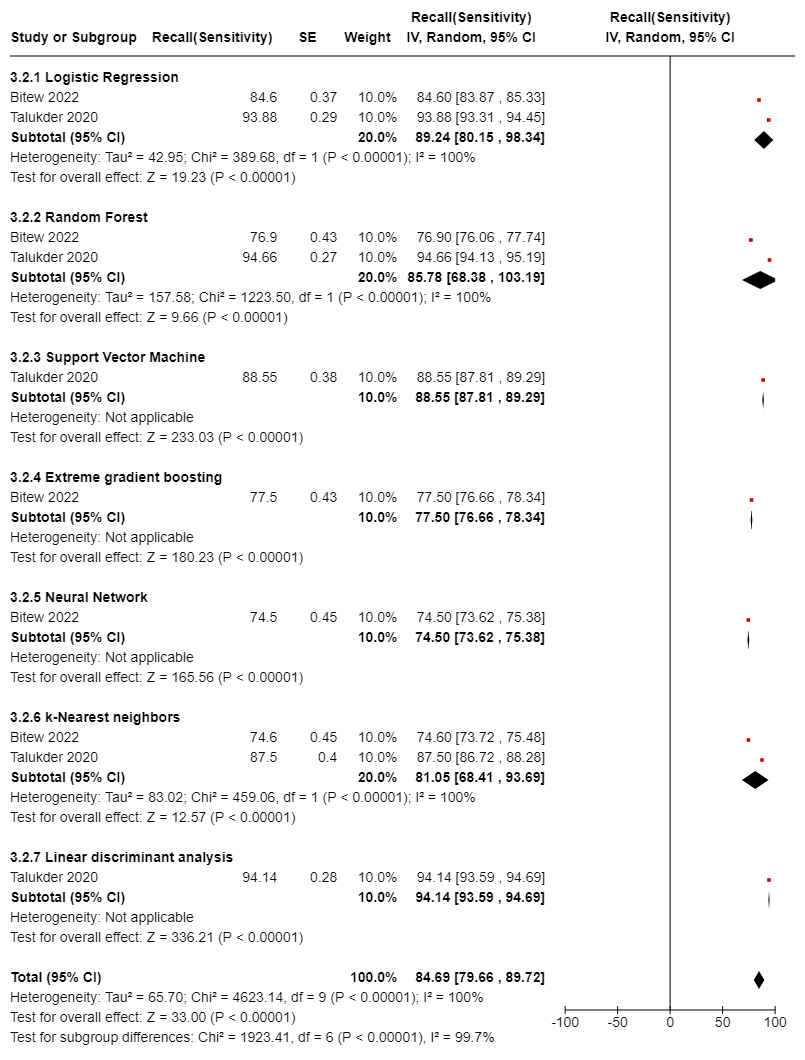

Supplement: Supplementary file 1 [file ijerph-22-00449-s001.zip › File S9.png]
